# Copper Binding and Redox Activity of α-Synuclein in Membrane-Like Environment

**DOI:** 10.3390/biom13020287

**Published:** 2023-02-03

**Authors:** Chiara Bacchella, Francesca Camponeschi, Paulina Kolkowska, Arian Kola, Isabella Tessari, Maria Camilla Baratto, Marco Bisaglia, Enrico Monzani, Luigi Bubacco, Stefano Mangani, Luigi Casella, Simone Dell’Acqua, Daniela Valensin

**Affiliations:** 1 Department of Chemistry, University of Pavia, Via Taramelli 12, 27100 Pavia, Italy; 2 Department of Biotechnology, Chemistry and Pharmacy, University of Siena, Via Aldo Moro 2, 53100 Siena, Italy; 3 Department of Biology, University of Padova, 35121 Padua, Italy; 4 Study Center for Neurodegeneration (CESNE), 35121 Padua, Italy; 5 CIRMMP, Via Luigi Sacconi 6, 50019 Sesto Fiorentino, Italy

**Keywords:** copper(II), copper(I), synuclein, redox activity, membrane environment, α-helix

## Abstract

α-Synuclein (αSyn) constitutes the main protein component of Lewy bodies, which are the pathologic hallmark in Parkinson’s disease. αSyn is unstructured in solution but the interaction of αSyn with lipid membrane modulates its conformation by inducing an α-helical structure of the *N*-terminal region. In addition, the interaction with metal ions can trigger αSyn conformation upon binding and/or through the metal-promoted generation of reactive oxygen species which lead to a cascade of structural alterations. For these reasons, the ternary interaction between αSyn, copper, and membranes needs to be elucidated in detail. Here, we investigated the structural properties of copper-αSyn binding through NMR, EPR, and XAS analyses, with particular emphasis on copper(I) coordination since the reduced state is particularly relevant for oxygen activation chemistry. The analysis was performed in different membrane model systems, such as micellar sodium dodecyl sulfate (SDS) and unilamellar vesicles, comparing the binding of full-length αSyn and *N*-terminal peptide fragments. The presence of membrane-like environments induced the formation of a copper:αSyn = 1:2 complex where Cu^+^ was bound to the Met1 and Met5 residues of two helical peptide chains. In this coordination, Cu^+^ is stabilized and is unreactive in the presence of O_2_ in catechol substrate oxidation.

## 1. Introduction

αSynuclein (αSyn) has been identified as the primary component of proteinaceous fibrillary deposits, known as Lewy bodies (LB), which represent the histological hallmarks of Parkinson’s disease [1]. αSyn is an intrinsically disordered 14 kDa protein, mainly localized in proximity to the presynaptic membranes and is directly involved in the physiological recycling of neurotransmitter vesicles and in dopamine metabolism [2]. Relatively recent works suggest that αSyn acts as a chaperone which facilitates the SNARE complex reassembly needed for vesicle–membrane fusion and for neurotransmitter release [3]. An amphipathic *N*-terminus 1–60 is required for neuronal membrane binding while a highly hydrophobic sequence, called a NAC domain (residues 61–95), regulates the fibrillation process [4,5]. Upon the electrostatic interaction with neuronal phospholipids, the protein is subjected to structural changes from an unstructured state to a helical conformation, mainly involving the *N*-terminal and NAC regions [6]. In the membrane-anchored form, the oligomerization of the protein is slower such as its propensity to generate metal complexes. Indeed, the protein takes up different metal ions with modest/high affinities, but only the long-term exposure to copper ions is effective in promoting fibrillation [7]. Although the αSyn aggregation mechanism is still an ambiguous theme, the dysregulation in metal homeostasis plays an important role in the progression of several ageing neurodegenerative disorders, included Parkinson’s [8,9]. In the *N*-terminal region of the protein, two binding sites have been identified for copper(II) interaction [10]. Site 1, confined in the residues 1–9, corresponds to a high affinity binding site (K_d_ from 10^−7^ to 10^−10^ M) and coordinates copper(II) ion via the involvement of the NH_2_ group of Met1, the amide group and the carboxylate side chain of Asp2, and one water molecule. In Site 2, the metal ion is anchored with low affinity (K_d_ from 10^−5^ to 10^−6^ M) through the participation of the amide and the imidazole groups of His50 together with the amide group of Val49 and a water ligand [7]. An additional low affinity copper(II) binding site centered around Asp121 was recently proposed to increase αSyn aggregation tendency [11]. However, several studies suggest that the *N*-terminal Met is acetylated in vivo [12], implying that copper(II) binding is lost after this post-translational modification [13]. Moreover, the protein localization in membranes strongly affects the metal coordination chemistry, by confining the histidine-50 residue (a relevant Cu^2+^-ligand in cytoplasmatic *medium*) in a phospholipid-interacting helical structure, thus precluding the simultaneous coordination of this residue with the *N*-terminus [14]. The characterization of the binding of copper in the reduced state shows a different coordination mode for αSyn. The presence of two conserved -M(X)_n_M- motifs, located at the *N*- and *C*-termini, provides a stable coordination shell for copper(I) binding via the thioethers of Met1/Met5 and Met116/Met127, respectively [15,16,17,18]. Investigations of the *N*-terminal coordination sphere for copper(I) ions performed by EXAFS and NMR approaches have revealed a tetrahedral disposition of 2S2O/N ligands around the cuprous ion [19]. On the other hand, only the involvement of Met1 and Met5 has been demonstrated, while the identification of oxygen/nitrogen donor residues is still debatable. NMR structural calculations have attributed the potential involvement as Cu^+^-anchoring ligands to the carboxylate group of Asp2 and a water/acetonitrile molecule.

The ability of copper bound to αSyn to catalyze redox reactions, such as catechol and ascorbate oxidation, has been largely proposed [10]. In the presence of neuronal reducing molecules such as catecholamines, copper(II) can be reduced to copper(I) species, resulting in reactive oxygen species (ROS) production during the metal redox cycling which contributes to the neuronal oxidative stress and related damage observed in PD [20]. Copper binding to neuronal peptides can alternatively promote (i.e., copper–amyloid-β [21], copper–tau [22], or copper–prion [23] fragments complexes) or reduce this oxidative reactivity [24], as in the case of copper–αSyn complexes [25].

Given the importance of αSyn–membrane interactions, it is therefore crucial to clarify how this interaction affects copper–αSyn binding mode and redox reactivity. Previous works have described how the interaction of the Cu–αSyn *N*-terminal fragment (1–15) complex with SDS micellar structures can alter the coordination chemical environment of the metal, thus affecting its Cu^2+^/Cu^+^ redox cycling [26]. In particular, the conformational change to an α-helical structure induced by SDS gives rise to a coordination of two peptide fragments to a single copper ion yielding a tetrahedral CuS_4_ site through the side chains of the *N*-terminal methionines. Moreover, the affinity of this complex is higher than that obtained in aqueous solution.

In order to clarify the redox potential of the copper–αSyn complex, it is essential first to obtain a more extensive characterization of the coordinative shells provided by the protein for both metal oxidative states, in free as well as in membrane-bound forms. A recent study showed that copper(II) modulates the interaction between αSyn and membrane-like matrices (lipidic cubic phase), but the role of copper(I) was not investigated [27]. Therefore, we aim to delineate the copper binding environment (with particular interest on coordination shell and on binding specificity of Cu^+^ ions) in full-length αSyn and in some synthetic peptide models through NMR, EPR, and XAS analyses together with the redox activity studies of the resulting metal complexes promoted by the presence of catechol substrates. In order to assess how the association of metal–protein complexes with lipid assemblies may affect copper redox chemistry, the studies have been performed in membrane model systems, such as micellar sodium dodecyl sulfate (SDS) and unilamellar vesicles (UVs).

## 2. Materials and Methods

### 2.1. Protein and Reagents

Human wild-type αSyn cDNA, amplified by Polymerase Chain Reaction (PCR) with synthetic oligonucleotides (Sigma-Aldrich) containing NcoI and XhoI restriction sites, was cloned into a pET-28a plasmid (Novagen, Merck KGaA, Darmstadt, Germany). Then, the protein was expressed in *Escherichia coli* BL21 (DE3) cells. Overexpression of proteins was achieved by growing cells in LB *medium* or, for the ^15^N-labeled protein, in M9 minimal *medium* prepared with ^15^NH_4_Cl (CIL, Cambridge Isotope Laboratories, Inc., Andover, MA), at 37 °C until an optical density at 600 nm (OD600 nm) of 0.3–0.4 was reached, followed by induction with 0.1 mM isopropyl β-thiogalactopyranoside (IPTG) for 4–5 h. The cells were collected by centrifugation and recombinant protein was recovered from the periplasm by osmotic shock as previously described [28]. The periplasmic homogenate was then boiled for 15 min and the soluble fraction containing αSyn, was subjected to a two-step (35% and 55%) ammonium sulfate precipitation. The pellet was then resuspended, extensively dialyzed against 20 mM Tris-HCl pH 8.0, loaded into a 6 mL Resource Q column (GE Healthcare, Fairfield, CT), and eluted with a 0–500 mM gradient of NaCl. Finally, the protein was dialyzed against water, lyophilized, and stored at −20 °C. The resulting protein had an unmodified α-NH_2_ terminal.

The *N*-terminal αSyn_1–15_ peptide was synthesized in solid phase using Fmoc chemistry. Rink amide resin was used as the solid support, so that the resulting peptides would be amidated at the *C-*terminus. After removal of the peptide from the resin and deprotection, the crude product was purified by RP HPLC on a Phenomenex Jupiter Proteo C12 column, using a Jasco PU-1580 instrument with diode array detection (Jasco MD-1510), with a semi-linear gradient of 0.1% trifluoroacetic acid (TFA) in water to 0.1% TFA in CH_3_CN over 40 min. The identity of the peptide was confirmed by Electrospray ionization mass spectrometry (Thermo-Finnigan). The purified peptide was lyophilized and stored at −20 °C until use. The αSyn_45–55_ peptide was purchased from Biomatik.

### 2.2. Unilamellar Vesicle Preparation

Liposomes were prepared mixing Phosphatidyl Choline (POPC) 70%, and Phosphatidyl Glycerol (POPG) 30% to reproduce the same conditions adopted by Dudzik et al. for investigating the αSyn–copper(II) interaction in membranes [29]. Both POPC and POPG lipids at the desired molar ratio were dried down from chloroform stock solutions under a stream of nitrogen gas and then dried under vacuum for 1 h. The resulting lipid film was hydrated by adding buffered solutions at physiological pH. Unilamellar vesicles (UVs) were prepared by freeze–thawing this lipid suspension five times followed by extrusion through 200 or 40 nm polycarbonate membrane filters using a mini-extruder syringe device (Avanti Polar Lipids).

### 2.3. NMR, EPR, XAS and CD Spectroscopic Measurements

Full-length αSyn protein was dissolved in 20 mM phosphate buffer in H_2_O at pH 7.4, obtaining a final concentration of 240 μM for NMR experiments, 500 μM for EPR experiments, and 10 μM for CD experiments. The protein concentration was estimated by spectrometry using a molar extinction coefficient at 274 nm of 5960 M^−1^ cm^−1^ [30]. The peptides were dissolved in 20 mM phosphate buffer aqueous solution at pH 7.4, obtaining a final concentration ranging from 400 to 500 μM for NMR and EPR experiments and 100 μM for CD experiments. A total of 50 mM SDS was added to both the full-length αSyn and the synthetic fragments solutions. The desired concentration of Cu^2+^ and Ag^+^ ions was achieved by using stock solutions of CuSO_4_ and AgNO_3_ (Sigma Chemical Co., St. Louis, MI, USA), respectively, in D_2_O. TMSP-2,2,3,3-d_4_, 3-(trimethylsilyl)-[2,2,3,3-d_4_] propansulfonate, sodium salt, was used as an internal reference standard for the NMR measurements.

CD spectra were acquired on a Jasco J-815 spectropolarimeter at 298 K. A 0.1 cm cell path length was used for data between 180 and 260 nm, with a 1 nm sampling interval. Four scans were collected for every sample with a scan speed of 100 nm min^−1^ and bandwidth of 1 nm. Baseline spectra were subtracted from each spectrum and the data were smoothed using the Savitzky–Golay method [31]. Data were processed using the Origin 5.0 spread sheet/graph package. The direct CD measurements (θ, in millidegrees) were converted to mean residue molar ellipticity, using the relationship mean residue Δε = θ/(33,000 × c × l × number of residues), where c and l refer to molar concentration and cell path length, respectively.

NMR spectra were acquired at 298 K using Bruker Advance spectrometers operating at proton frequencies of 600 MHz and 900 MHz, with the latter one equipped with a cryoprobe. The NMR spectra were processed with TopSpin 3.6 software and analyzed with the program Cara [32]. Suppression of residual water signal was achieved either by presaturation or by excitation sculpting [33], using a selective 2 ms long square pulse on water. Proton resonance assignment of the peptides was obtained by 2D ^1^H-^1^H COSY, TOCSY, and NOESY experiments. The metal interaction studies were performed by acquiring 2D ^1^H-^15^N HSQC and ^1^H-^13^C HSQC experiments.

EPR measurements (CW X-band (9.4 GHz)) were carried out with a Bruker Elexsys E500 series using the Bruker ER4122 SHQE cavity and an Oxford helium continuous-flow cryostat (ESR900). Low temperature spectra were simulated using a software for fitting EPR frozen solution spectra that is a modified version of the program written by J.R. Pilbrow (Cusimne) [34].

XAS samples consisted of 500 μM protein/peptide solutions with UVs or SDS micelles in 20 mM phosphate buffer at pH 7.4 in presence of 0.5 Cu^+^ eqs. (Cu^2+^: αSyn ratio of 1:2). The sample holder and the kapton windows had been previously washed carefully with a highly concentrated ethylenediaminetetraacetic acid (EDTA) solution (approximately 100 mM), rinsed with pure water and absolute ethanol, and dried. The sample cell was then mounted in a cryostat and kept at 100 K during data collection.

X-ray absorption near-edge structure (XANES) and extended X-ray absorption fine structure (EXAFS) data were collected at the GILDA CRG beamline of the European Synchrotron Radiation Facility (Grenoble, France) using a Si(311) double-crystal monochromator employing dynamical sagittal focusing. The photon flux was of the order of 10^10^ photons s^–1^ and 1 mm × 1 mm spot size. The XAS data were recorded by measuring the Cu Kα fluorescence using a Ge 12-element solid-state detector over the energy range from 8800 to 9600 eV using variable energy step widths. In the XANES and EXAFS regions, steps of 0.5 and 2.0 eV were used, respectively. Ionization chambers in front and behind the sample were used to monitor the beam intensity. Energy calibration of the spectra was obtained by measuring metallic Cu foil. Due to the quite low concentration of the absorber achievable in all samples, all the datasets were usable over a limited k range. This allowed the data analysis to be performed only to the first/second Cu^+^ coordination shell.

### 2.4. XAS Data Analysis

XAS data reduction and normalization were performed using the Athena module of the Demeter XAS software package, [35] whereas the extracted EXAFS spectra were analyzed by the Artemis module of the same software [35]. All data were fitted with k^3^ weighting over a k range of 3–8 Å^−1^ in case of αSyn_1–15_ SDS–Cu^+^ and αSyn UVs–Cu^+^, and 2–7.5 Å^−1^ for αSyn SDS–Cu^+^, with an R-space over a different r-range for a particular system (1–3.6 Å for αSyn_1–15_ SDS–Cu^+^, 1–3.0 Å for αSyn UVs–Cu^+^ and 1–3.4 Å for αSyn SDS–Cu^+^). The following parameters were determined during the fitting: the energy shift (ΔE_0_), the bond distances, and Debye–Waller factor (2σ^2^) for the first coordination shell. The passive electron reduction factor (S_0_^2^) was set to 0.90 for all the studied systems. The goodness of each fit was assessed by minimizing the values of the R-factor and reduced χ^2^ for fits with 2σ^2^ values in the range of 0.0015–0.019 Å^2^. The plots are S phase corrected.

### 2.5. Catalytic Oxidation of 4-Methyl Catechol and Dopamine by Cu^2+^ and Cu^+^ Ions Bound to the Full-Length αSyn

The catalytic oxidation of 4-methylcatechol (4-MC) and dopamine (DA) by copper(II) was studied at room temperature in 50 mM HEPES buffer at pH 7.4, saturated with atmospheric oxygen. The development of the relative quinone band at 401 nm for 4-MC and at 475 nm for DA was followed for 1800 s. The concentrations of copper(II) nitrate and substrate were fixed at 25 µM and 3 mM, respectively. The autoxidation was also evaluated, performing the experiment in the presence of substrate alone (3 mM) in buffer solution. The redox activity of Cu^2+^–αSyn complexes was evaluated at 1:2 or 1:1 molar ratios relative to copper, in the absence or upon the addition of 20 mM SDS.

In order to study the catalytic oxidation of 4-MC by Cu^+^ and Cu^+^–protein in micellar *medium*, copper(II) ions were anaerobically reduced to copper(I) (25 μM) by ascorbate (50 μM) in the presence of SDS (20 mM). Upon vacuum cycles, MC (3 mM) was added to the solution as it was rapidly exposed to air. The same experiment was repeated in the presence of the Cu^+^–αSyn adduct with a 1:2 molar ratio.

### 2.6. HPLC-ESI/MS Analysis of Protein Oxidative Modification

The solutions obtained from the kinetic data and incubated for different reaction times were quickly acidified and frozen. Each sample was prepared by adding copper(II) nitrate (25 µM), αSyn (50 µM), 4-MC (3 mM), and SDS (0–20 mM) in 50 mM HEPES buffer at pH 7.4. Before the HPLC-MS analysis of the SDS-containing samples, an excess of potassium chloride was added in each cooled-down reaction mixture in order to induce the detergent precipitation, which was then removed through centrifugation. The enzymatic cleavage was achieved by using αSyn:pepsin enzyme at 50:1 w/w and, after an incubation time of about 2 h and 30 min at 37 °C, all samples were analyzed by LC-MS.

## 3. Results and Discussion

### 3.1. Characterization of Cu^+^/Ag^+^ Binding to Membrane-Bound αSyn

First, the identity of Cu^+^ binding sites in the membrane-bound full-length protein was assessed by NMR spectroscopy. As demonstrated in previous works, NMR studies performed using silver(I) ions as a probe for copper(I) interactions with protein are functional in order to obtain redox-inactive complexes while preserving similar electronic and structural properties of copper(I) complexes [15,26]. The high affinity of silver(I) for soft donor ligands makes this ion particularly suited to investigate copper(I) binding site and copper metabolism while avoiding Fenton-like reactions [36,37].

**Figure 1:**
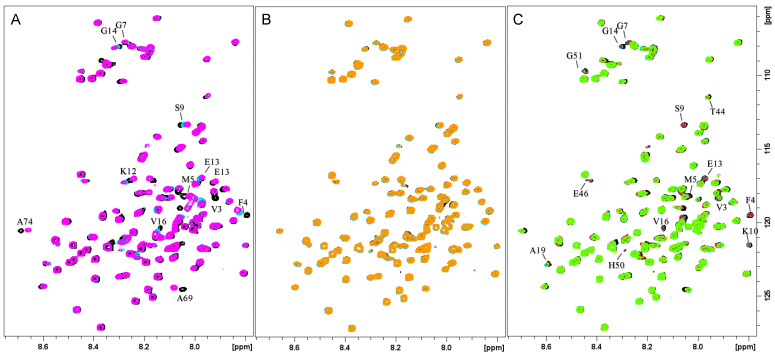
Overlaid ^1^H-^15^N HSQC spectra of micelle-bound αSyn_1–140_ (^15^N uniformly enriched) in the presence of increasing amounts of metal ion: (**A**) 0 eq (black), 0.3 Ag^+^ equiv. (light blue), 0.6 Ag^+^ equiv. (magenta); (**B**) 0.6 Ag^+^ equiv. (black), 0.8 Ag^+^ equiv. (light blue), 1 Ag^+^ equiv. (orange); (**C**) 0 eq (black), 0.2 Cu^2+^ equiv. (light blue), 0.4 Cu^2+^ equiv. (red), 0.6 Cu^2+^ equiv. (green). αSyn_1–140_ 240 μM, SDS-d_25_ 50 mM, phosphate buffer 20 mM pH = 7.4, T = 298 K.

The effects of silver ions on NMR resonances of αSyn were first evaluated by recording ^1^H-^15^N HSQC spectra at 298 K on uniformly ^15^N-enriched αSyn in the presence of SDS micelles. The metal-induced changes in the spectra were monitored by gradually increasing the amount of metal. When Ag^+^ ions were added to the protein solution, several chemical shift displacements were observed for both ^1^H and ^15^N frequencies belonging to Val3, Phe4, Met5, Gly7, Ser9, Lys12, Glu13, Gly14, and Val16 (Figure 1A). When similar experiments were performed in aqueous solution [15], major displacements of residue signals were observed at both the *N*- and *C*-termini of the protein, on proton and nitrogen resonances of amino acids encompassing the regions 1–5 and 121–127. On the contrary, when the protein was trapped by micellar structures with the largest effects resulting from Ag^+^ interactions localized at the *N*-terminus of the protein only. Similar conclusions were obtained by looking at ^1^H-^13^C HSQC NMR spectra (Figure S1), where metal binding caused large chemical shift variations only on Met1 and Met5 -SCH_3_ cross-peaks. The exclusive effects on Met1 and Met5 signals were also evident by comparing the metal-induced perturbations in the ^1^H-^13^C HSQC spectra of αSyn and the αSyn_1–15_ fragment lacking the Met116 and Met127 residues (Figure S1). All these findings indicate that only two out of the four methionine residues in αSyn are shifted by the metal ions, thus supporting (*i*) coordination to the *N*-terminal region and (*ii*) sulfur involvement in the metal coordination sphere. Finally, the analysis of 2D ^1^H-^15^N HSQC titration experiments acquired in the presence of increasing amounts of Ag^+^ ions showed that the chemical shift plateau was almost reached upon the addition of 0.6 Ag^+^ equiv. (Figure 1B). This behavior is consistent with a metal: αSyn ratio of 1:2 and indicates the formation of bis-complexes, in which Met1 and Met5 residues are detected as the silver binding region with the Met δ–sulfur atoms bound to Ag^+^. These results are fully in agreement to that obtained for the interaction between Ag^+^ and αSyn_1–15_ peptide [26], confirming that the *N*-terminal fragment is a suitable model for metal coordination and the use of full-length protein does not affect the metal binding. The silver binding site observed for αSyn was also conserved in acetylated αSyn as shown by the almost identical silver-induced NMR variations recorded for both *N*-terminus free and acetylated protein forms (Figure S2).

**Figure 2:**
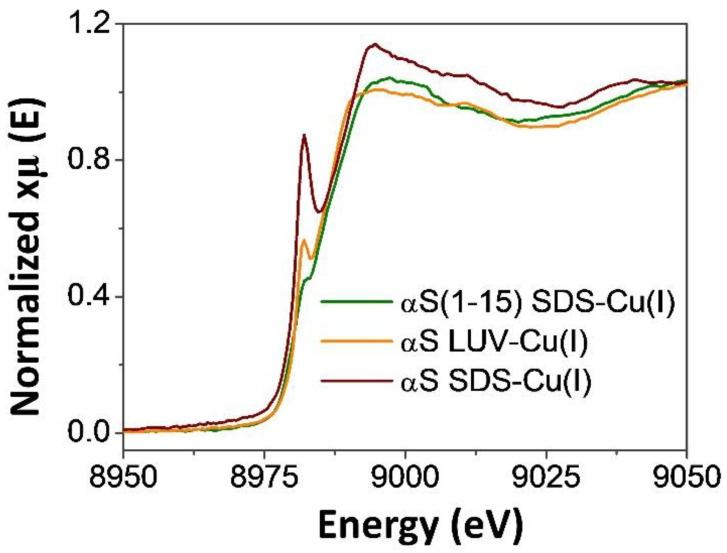
Cu K-edge XANES spectra of the membrane-bound αSyn_1–140_ and αSyn_1–15_ Cu^+^ complexes. αSyn_1–140_ 500 μM, αSyn_1–15_ 500 μM, Cu^2+^ 250 μM, SDS 50 mM, 125:1 lipid:protein ratio, phosphate buffer 20 mM pH = 7.4.

The coordination sphere of Cu^+^ in model membranes-bound αSyn was additionally investigated by XAS spectroscopy. The studied systems included the model peptide αSyn_1–15_ in the presence of SDS micelles (αSyn_1–15_ SDS-Cu^+^), the full-length protein in the presence of SDS micelles (αSyn SDS-Cu^+^), and in the presence of unilamellar vesicles (αSyn UVs-Cu^+^), which are known to behave as a more realistic membrane model. XANES spectra (Figure 2) revealed a pre-edge feature at about 8982 eV characteristic for Cu^+^ complexes, which can be interpreted as Cu^+^ 1s-4p transition [35]. As shown in Figure 2, different peak intensities were measured according to the investigated systems. The lowest intensities were observed for αSyn_1–15_ SDS-Cu^+^ and αSyn UVs-Cu^+^, whereas the intensity of αSyn SDS-Cu^+^ was significantly higher. Considering that the intensities of the XANES peaks are strongly dependent on the geometry and coordination number of the metal complex [35,38,39], our data indicate that the αSyn_1–15_ SDS–Cu^+^ and αSyn UVs–Cu^+^ complexes are very similar; on the other hand, the differences exhibited by the αSyn SDS–Cu^+^ complex point out local rearrangements of the copper binding sites when the protein is bound to SDS.

**Figure 3:**
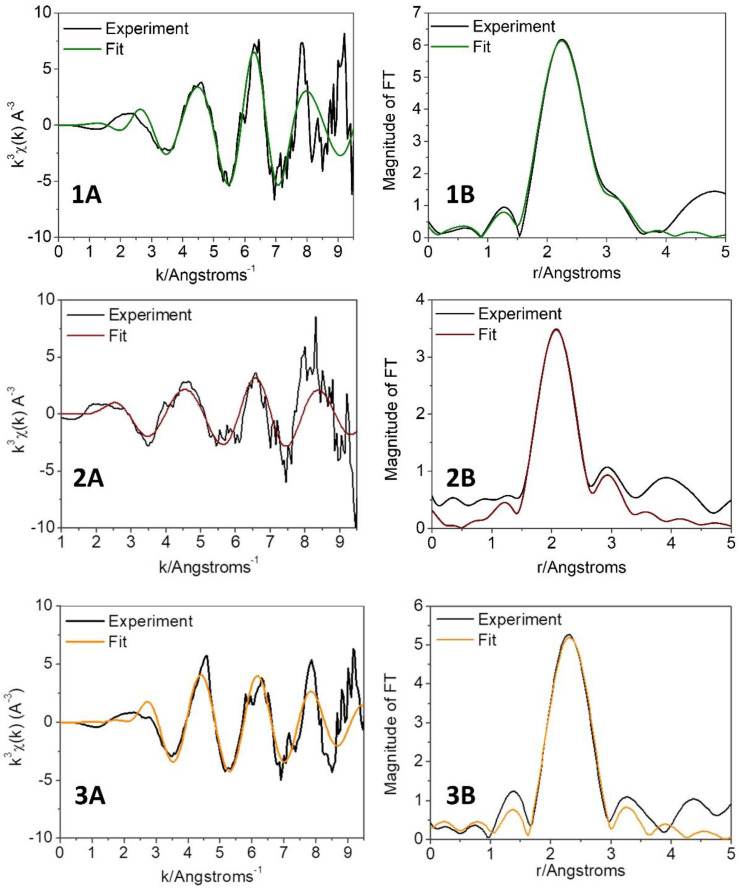
Experimental and simulated k^3^ weighted EXAFS spectra of αSyn_1–15_ SDS-Cu^+^ (**1A**), αSyn SDS-Cu^+^ (**2A**), and αSyn UVs-Cu^+^ (**3A**), and Fourier transform of αSyn_1–15_ SDS-Cu^+^ (**1B**), αSyn SDS-Cu^+^ (**2B**), and αSyn UVs-Cu^+^ (**3B**) spectra. αSyn_1–140_ 500 μM, αSyn_1–15_ 500 μM, Cu^2+^ 250 μM, SDS 50 mM, 125:1 lipid:protein ratio, phosphate buffer 20 mM pH = 7.4.

To get more insight into the metal coordination sphere, the EXAFS regions of all three systems were further analyzed. The fitting models including four, three, and two sulfur atoms and the possible presence of a N/O atom in the coordination sphere were also investigated. The experimental and theoretical k^3^ weighted EXAFS spectra and Fourier transforms of the best fitting models are shown in Figure 3, while the corresponding parameters are listed in Table 1. Examples of other relevant fitting models are shown in Table S1.

EXAFS analysis pointed out the preference of copper(I) coordination to four sulfur atoms for all the three investigated systems, independently of the membrane model used. These findings strongly agree with the NMR data recorded for the αSyn SDS–Ag^+^ complex (*vide supra*) and the one previously reported on Ag^+^/Cu^+^ interactions with αSyn_1–15_ peptide [26]. However, the Cu–S distances shown in Table 1 indicate that, according to the membrane model, metal donor atoms adopt different coordination rearrangements. The reported set of Cu^+^–S distances (two short (2.16/2.12 Å) and two long (2.68/3.00 Å)) obtained for αSyn_1–15_ SDS–Cu^+^ and αSyn SDS–Cu^+^ are rather uncommon for a tetrahedral Cu^+^–Met complex [40,41], although such Cu^+^–S distances have been identified in metal centers of some proteins (e.g., in plastocyanins and rusticyanin) [40,42]. Moreover, the association of the peptides with membranes, resulting in conformational changes to α-helical structures, may lead to some limitations in the orientation of methionine side chains. In multicopper oxidase CueO, where Cu^+^ ions are bound by Met-rich helical region, various Cu^+^–S(Met) distances were observed [41]. Additionally, the EXAFS fitting results of αSyn_1–15_ SDS–Cu^+^ are significantly improved by adding eight carbon atoms, corresponding to γ and ε carbon atoms of 4 Met (see Fit 1 in Table S1). The model including 4 S atoms at the same distances led to a 3-fold increase of the R-factor (Fit 2 in Table S1). Even better results were obtained for the model where 3 S at the same distances were bound to Cu^+^ (see Fit 4 in Table S1); however, this fit produced parameters that are less satisfactory compared to the model with 2 short and 2 long Cu^+^–S distances. In the literature, model lipid membranes composed of synthetic phospholipids such as POPC/POPS, were extensively used to better mimic the protein–membrane interaction. Herein, we have additionally performed quantitative EXAFS analysis on αSyn–Cu^+^ systems in the presence of negatively charged unilamellar vesicles and the data revealed 4 S atoms located at 2.28 Å in the first coordination shell, where the obtained Cu^+^–S distances are typical for tetrahedral copper–sulfur complexes [42,43]. However, they were slightly shorter than those observed in CopK proteins, where Cu^+^ is bound by 4 Met residues with an average distance of 2.31 Å [44].

To explain the different copper–sulfur distances pointed out by the XAS data, it is fundamental to consider the protein/peptide structural rearrangements occurring upon SDS/UVs–αSyn interactions. In fact, it is known that both systems can induce α-helix structures whose features are strongly dependent on the type of the membrane model system [45,46,47,48,49]. Moreover, it should be considered that copper(I) binding to two peptide/protein units (bis-complexes) requires the close proximity of two α-helix synuclein molecules simultaneously interacting with SDS/UVs. According to that, our data identified UVs as a more appropriate environment for the formation of a tetrahedral Cu^+^–αSyn complex, contrary to SDS forcing Cu^+^ to adopt a more distorted geometry.

### 3.2. Characterization of Cu^2+^ Binding to Membrane-Bound αSyn

Although copper(II) binding with αSyn has been extensively elucidated in aqueous *medium*, little data of the structural and catalytic properties of the metal complexes in membrane-like systems are available. Indeed, the protein’s ability to chelate both copper redox states is a necessary condition for ROS generation and for fibrillar deposition into insoluble aggregates. The presence of lipid assembly affects both the oligomerization state of αSyn and [Cu^+^/Cu^2+^–protein] redox cycling, which may be the result of alterations in metal coordination mode and affinity.

The impact of copper binding to micelle-bound αSyn was first evaluated by looking at the far-UV CD spectra of the full-length protein recorded in the absence and presence of increasing amounts of Cu^2+^ ions and 50 mM SDS. The spectra showed the unvaried presence of the two negative bands at 206 and 222 nm, that are characteristic of the α-helical structure (Figure S3) and suggest that the binding of Cu^2+^ to the protein does not affect the α-helical structure adopted by the first 100 residues in the presence of SDS micelles [50,51,52]. These findings are in full agreement with those previously reported by Dudzik and collaborators, who showed that the interaction of αSyn with Cu^2+^ ions in the presence of small unilamellar vesicles does not affect the α-helical content [29]. On the other hand, previous investigations found an α-helix enhancement induced by copper binding to membrane-bound αSyn [29,53].

To identify the copper(II) binding regions in lipid-bound αSyn, Cu^2+^ binding to the full-length protein and its derived model peptides, αSyn_1–15_ and αSyn_45–55_ (the two αSyn regions usually affected by the paramagnetic ion in aqueous solutions) was investigated. The analysis of Cu^2+^ interactions was performed in the presence of SDS micelles by means of NMR, EPR, and CD spectroscopy. Comparison of the ^1^H-^15^N HSQC spectra of full-length αSyn in the absence and presence of increasing amounts of Cu^2+^ allowed the identification of the Cu^2+^-binding regions in the protein (Figure 1C). After addition of copper(II) ions, selective line broadening effects due to paramagnetic relaxation rate enhancement were observed, mainly localized at the regions encompassing the residues 1–16 and 46–52. In particular, cross-peaks belonging to Val3, Phe4, Gly7, Ser9, Lys10, Glu13, Gly14, Val 16, Glu46, His50, and Gly51 were completely washed out by the addition of 0.6 Cu^2+^ equiv. No effects were detected on the αSyn *C*-terminal region, contrary to what was recently observed by in-cell NMR measurements of Cu^2+^ interactions with αSyn expressed in the *E. coli* periplasm, that is known to associate with cellular membranes [27]. Such behavior might be explained by considering the diverse copper concentrations used in the two studies, the latter being more than one order of magnitude larger and thus justifying the effects detected on the low affinity copper binding site.

**Figure 4:**
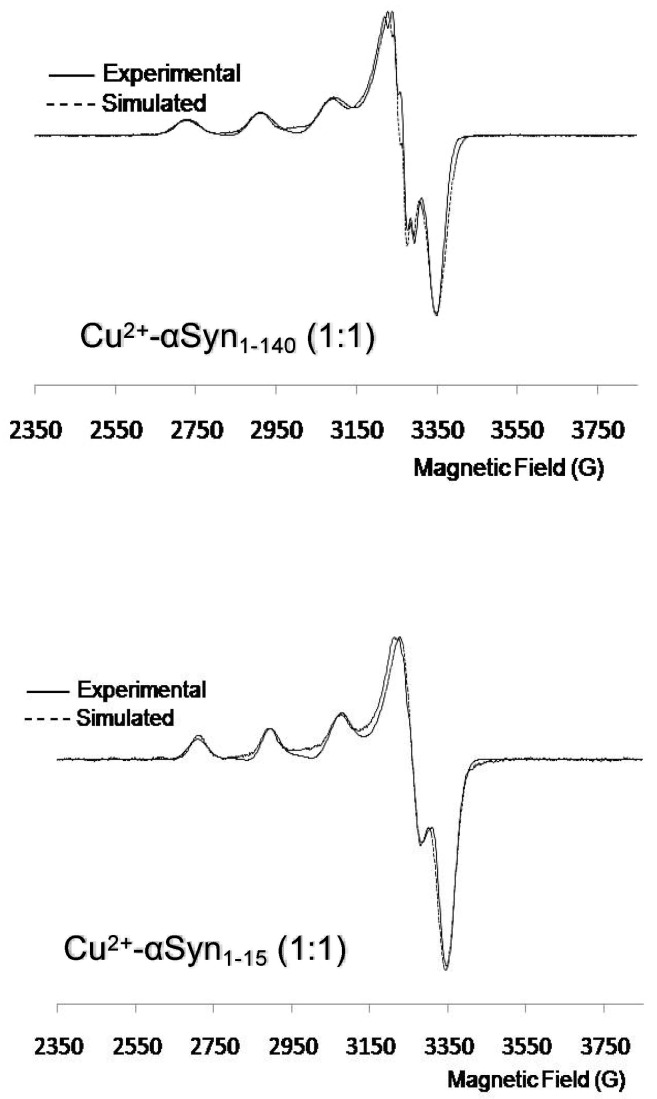
EPR spectra of micelle-bound synuclein Cu^2+^ complexes: αSyn_1–140_ (**upper panel**) and αSyn_1–15_ (**lower panel**). αSyn_1–140_ 500 μM, αSyn_1–15_ 500 μM, Cu^2+^ 500 μM, SDS 50 mM, phosphate buffer 20 mM pH = 7.4.

With the aim of obtaining more insight on the coordination sphere of the metal ion, the X-band EPR spectra of the Cu^2+^–peptide/protein systems at low temperature (70 K) were recorded. The superimpositions of the experimental and simulated spectra for Cu^2+^-αSyn_1–140_ and Cu^2+^-αSyn_1–15_ are shown in Figure 4, reporting the obtained anisotropic parameters in Table S2. The results with the full-length protein suggest the presence of an axial geometry around the metal coordination, supporting the formation of 2N species. Similarly, the EPR spectra obtained for Cu^2+^–αSyn_1–15_ complex show matching fine structure, suggesting similar coordination environment for Cu^2+^ ion, as also confirmed by the similarity of the obtained anisotropic parameters (Table S2). On the other hand, a completely different behavior was observed for Cu^2+^–αSyn_45–55_ (Figure S4), which closely resembled the one obtained for the blank solution containing only free metal ions. These findings are in agreement with already published EPR study and support that the *N*-terminal region is the only binding site in α-helix structured αSyn, while the region 45–55 functions as a very low affinity Cu^2+^ binding site [29].

An NMR investigation on Cu^2+^–αSyn_1–15_ complex was also carried out with the aim of identifying the minimal copper(II) binding site in folded αSyn. Copper(II)-induced line broadening on the peptide was monitored by analyzing the 2D ^1^H-^1^H TOCSY and ^1^H-^13^C HSQC maps (Figure S5). The more affected resonances belonged to Met1, Asp2, Val3, Phe4, and Met5 residues indicating the region 1–6 of the peptide as the Cu^2+^-binding domain. By comparing these effects with those previously obtained in the absence of detergent micelles [7,54,55], the same binding mode can be hypothesized for the system 1–15 in water solutions and in the membrane mimicking environment used in the present work, with the 2N donors identified by EPR analysis reasonably being the free Met1 amino group and the adjacent Asp2 amide group (α-NH_2_, N^−^).

Finally, CD studies on the ternary Cu^2+^–Ag^+^–αSyn_1–15_ system allowed us to evaluate the competitive binding of both copper redox states to the micelle-bound form of αSyn_1–15_. The addition of copper(II) to the solution containing αSyn_1–15_ peptide in SDS micelles caused the formation of a negative CD band at 305 nm due to LMCT transitions that occur in Cu^2+^ complexes (Figure S6). The intensity of the band increased as the metal concentration increased up to 0.55 Cu^2+^ equivalents. Further addition of Cu^2+^ ion did not affect the spectrum. When 1 Ag^+^ equivalent was added to the solution containing the Cu^2+^–αSyn_1–15_ complex, part of Cu^2+^ ion bound to the peptide was replaced by Ag^+^ and the intensity of the CD band decreased, reaching a value corresponding to a Cu^2+^ concentration between 0.1 and 0.35 equivalents, suggesting the ability of αSyn_1–15_ to simultaneously bind both metal ions.

### 3.3. Oxidative Reactivity of Copper–Full-Length αSyn Complex in Membrane Environment

The previous structural data have identified some divergences in copper(I) binding affinity when the protein assumes a helical conformation, while the copper(II) binding site seems to be less affected by the micelle-anchored state, suggesting that the Cu^2+^-binding region is trapped by the membrane and does not involve His50 in the coordination shell. From these considerations, we may speculate that metal redox cycling can be influenced by the association of the protein with a lipid assembly. Indeed, when copper(II) ions interact with αSyn protein in aqueous *medium*, the resulting complexes can be reduced to generate Cu^+^-species by cellular reductants, e.g., ascorbic acid or glutathione. Subsequently, the copper(I) complex reacts with molecular dioxygen to give ROS production and re-oxidation of the metal system.

To better clarify the metal redox cycling upon binding with membrane-anchored αSyn protein, we performed kinetic UV-Vis studies of oxidative reactivity toward some catechol substrates, as 4-methylcatechol and dopamine, catalyzed by the presence of the metal complex in lipid environment. The oxidation of the substrate (3 mM) promoted by copper was studied at 20 °C in 50 mM HEPES buffer at pH 7.4 and the metal was added at a concentration of 25 µM to the substrate solution in the presence of αSyn (0–50 µM). We compared the catalytic profiles obtained with Cu–protein complexes that were free in solution or SDS-anchored. The kinetic traces of 4-methylcatechol oxidation evidenced strong redox activity quenching of the [Cu–αSyn] complex (1:2) bound to the membrane-like system (Figure S7, left panel). Similar results were previously observed with a [Cu–PrP_76–114_] system, in which two vicinal Met residues, _109_MKHM_112_, were contained in the prion sequence analogously to αSyn protein [23]. We can suppose that these related domains in PrP and αSyn are the regions directly involved in Cu^+^ stabilization, where the Met-mediated coordinative environment makes the reduced metal ion highly unreactive to dioxygen. On the other hand, whereas in the Cu^+^–PrP_76–114_ system the metal binding occurs intramolecularly and involves one His residue (H_111_) besides the two methionines, in the Cu^+^–αSyn complex, the stabilization of the reduced ions is provided by the coordination of four Met residues belonging to the *N*-terminal regions of two αSyn proteins. The relationship between the generation of the Cu^+^(αSyn)_2_ complex and the reactivity shutdown is supported by the data obtained with the metal complex at a ratio of 1:1 (Figure S7, right panel). The reactivity in aqueous *medium* did not significantly diverge from the kinetic traces obtained with copper alone while the addition of detergent micelles only slightly affected the substrate oxidative reaction, suggesting lower Cu^+^ stabilization is provided by one bound protein.

Analogous oxidative trends were observed with substrate substitution of 4-MC by a catecholamine (dopamine, see Figure S8), suggesting that micelle-bound Cu–αSyn complexes were unreactive to dioxygen binding independently of the substrate reduction potential. Moreover, the different distribution of substrate between the aqueous and lipid phases has to be considered due to the electrostatic interactions between the anionic micellar surface and the amine group of dopamine, which influence the resulting catalytic efficiency of the Cu–αSyn complex.

**Figure 5:**
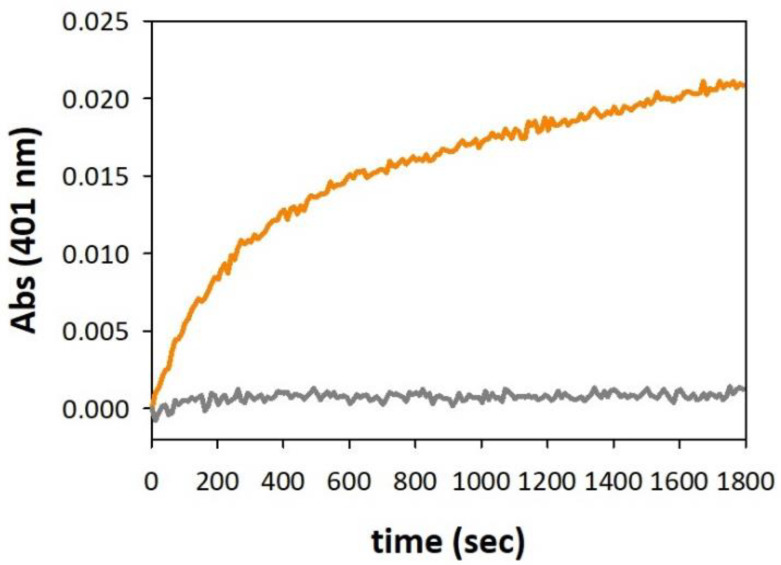
Kinetic profiles of 4-MC (3 mM) oxidation over time in 50 mM HEPES buffer at pH 7.4 and 20 °C containing SDS (20 mM) and in the presence of Cu^+^ (25 μM) alone (orange trace) or Cu^+^ (25 μM) and αSyn (50 μM) (grey trace). Copper(I) was generated in situ by anaerobic reaction of copper(II) nitrate (25 μM) and ascorbate (50 μM) prior to exposure of the solution to air.

The involvement of bis-protein Cu^+^ intermediate as a species inert to oxidation was also confirmed by UV-Vis kinetics carried out with the anaerobically prepared Cu^+^–αSyn complex (1:2). The anaerobic solution of catalyst was previously purged with pure argon and was simultaneously exposed to the substrate and to the atmospheric oxygen to follow substrate oxidation induced by the reduced metal–protein system. Unlike “free” copper(I), the oxidative reactivity of the Cu^+^–αSyn system trapped in the micellar environment resulted in total quenching (Figure 5), which agrees with the data obtained from Cu^+^–αSyn_1−15_ and Cu^+^–PrP complexes [23,26].

A recent study by Calvo et al. reported that the presence of a membrane environment increases the oxidation rate of dopamine promoted by copper–αSyn complexes [56]. However, this conclusion is in partial agreement with the data. In particular, the Vmax value obtained in the presence of the membrane was indeed two orders of magnitude higher than in its absence, but this increase was also obtained for the Vmax obtained with free copper. This comparison indicates that the increase in reactivity is due to the experimental conditions of the assay. Indeed, these results were probably affected by the use of 3-methyl-2-benzothiazolinone hydrazine (MBTH) as the reducing dye. MBTH is a strong and non-physiologically relevant reducing agent that allows a better visualization of the product formation, but its high and uncontrolled reactivity can alter the reaction mechanism.

### 3.4. HPLC-ESI/MS Analysis of Protein Oxidative Modification

The complexity of the redox properties of these systems was indirectly investigated as the result of ROS species production and therefore, oxidative damage on the protein itself. As previously described, the Cu(II)/(I) redox chemistry generates several oxidizing Cu/O_2_ species and, unlike the site-specific activity of the biological enzymes, the resulting reactivity of these systems is totally uncontrolled and leads to the production of oxidative agents, as hydroxyl radicals or hydrogen peroxide, that are able to attach and modify biological targets including αSyn itself.

We have characterized and partially quantified by HPLC-MS the amount of oxidative modification of αSyn upon its incubation for different reaction times in the presence of 4-MC (3 mM), cuprous/cupric ions (25 μM), and with/without SDS (0–20 mM). At the end of the incubation, all samples were proteolytically digested by adding pepsin (at 50:1 *w/w* αSyn: pepsin) and the modification pattern of the resulting fragments was then evaluated. The pepsin-mediated cleavage of the protein was achieved by working in strongly acidic *medium* conditions (pH ~ 1). In addition to the previous treatment, in SDS-containing samples the detergent micelles were extracted before enzymatic digestion. The precipitation of SDS was carried out as described in previous works by adding potassium chloride to each sample and removing the insoluble fraction through centrifugation of cold samples [57].

Although dopamine is the most biologically relevant catechol used in this work, the HPLC-MS quantification of the modified αSyn fragments was performed using 4-MC for reducing the heterogeneity of substrate oxidative products released in solution. As proposed in previous studies [23,25], we considered His and Met residues as the main ROS-mediated modification targets by taking into account their proximity to the redox-active copper ions. The αSyn modification pattern obtained from the sample incubated for 30 min in the presence of substrate and copper(II) ions allows the identification of several oxidation sites at both the *N*- and *C-*termini and characterized by mass increment of +16 amu (insertion of one *O*-atom) (see Figure S9). Moreover, by increasing the reaction time until 4 h, we observed more complex modification patterns both as an enhancement in relative percentage of oxidized residues as well as in the chemical nature of the modification, with an additional 21% covalent attachment of catechol/quinone molecules to the imidazole group of His50 (Table 2).

The analysis of the oxidative modifications of the protein when the reaction mixture contained SDS micelles showed that the redox quenching or low reactivity of these copper(I)/(II)-complexes, respectively, evidenced in the kinetic studies carried out in surfactant mixtures, are accompanied by a change in the protein modification percentages. Moreover, any relevant oxidative modification was detected when the catalyst of the oxidative reaction is the Cu+–αSyn complex trapped in SDS, which agrees with the total redox shutdown shown in Figure 5. On the other hand, this effect is rather less pronounced when we start from the oxidized catalyst, thus resulting in appreciable percentages of sulfoxide and 2-oxo-histidine generation undergone by the Met5 and His50 residues, respectively. We can therefore appreciate relevant protective effects provided by the interaction of copper complexes with SDS and these results are in agreement with those previously obtained on other copper systems such as Cu–PrP and Cu–Aβ [57]. We can also verify the total redox inertia of micelle-bound Cu(I)–αSyn complexes as result of negligible ROS production and lack of protein modification.

## 4. Conclusions

**Figure 6:**
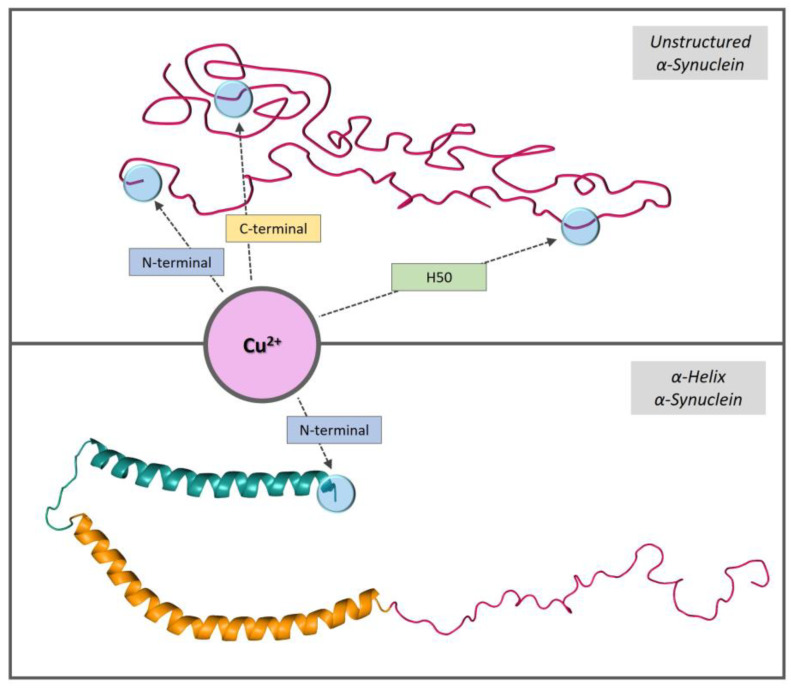
Schematic representations of αSyn copper(II)-binding sites in aqueous and membrane environments.

**Figure 7:**
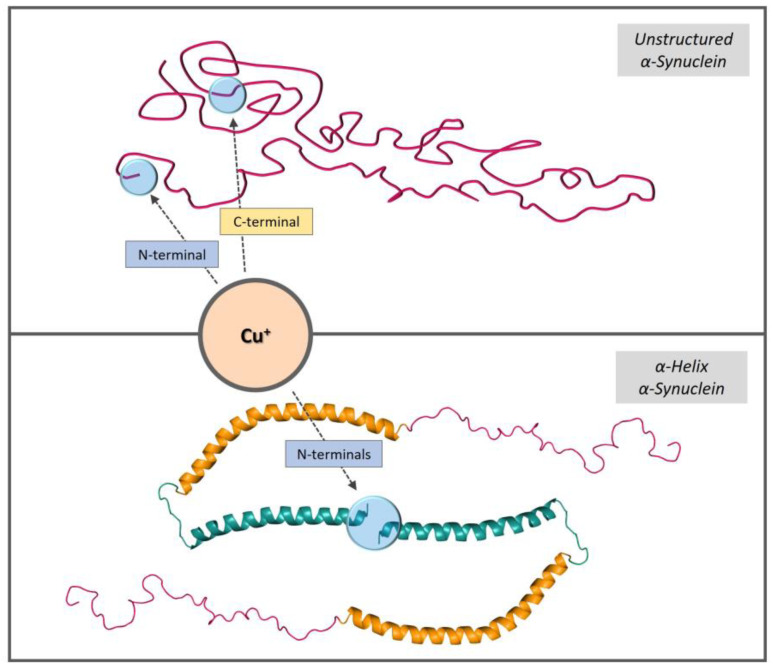
Schematic representations of αSyn copper(I)-binding sites in aqueous and membrane environments.

In this work, we investigated the binding of copper to αSyn and the redox activity of the relative complexes in a membrane-like environment. The interaction with membranes strongly affected αSyn structure by inducing a conformational change from unstructured to an α-helical structure in the *N*-terminal region. In this study, we used micellar sodium dodecyl sulfate (SDS) and unilamellar vesicles to mimic the membrane environment. NMR, EPR, CD, and XAS analyses of copper(I) and copper(II) binding revealed αSyn’s ability to modulate metal coordination according to the adopted conformation. In particular, the α-helical rearrangement stabilized copper(II) binding to the N-terminal region of the protein (Figure 6) and favored the formation of copper(I) bis-complexes, characterized by a tetrahedral CuS_4_ site through the side chains of the *N*-terminal Met1 and Met5 residues (Figure 7).

The oxidative reactivity of the resulting complex was explored in both oxidation of exogenous substrates such as dopamine and 4-MC, and/or with the protein bound to the metal. Both studies indicated a remarkable difference when the analysis was performed in solution or in the membrane-like environment. The quenched reactivity observed in the UV–visible kinetic analysis and the absence of relevant oxidative modification on the αSyn backbone in the HPLC-MS study indicated that the formation of the CuS_4_ site in the 1:2 copper:αSyn complex is relatively stable and retains the capacity to activate O_2_ and promote oxidative reactions.

These results confirm our previous data on the interaction between copper(I) and the 1–15 *N*-terminal fragment of αSyn in the presence of SDS as a membrane-like environment [26]. Herein, the same conclusion was obtained in the presence of the full-length protein and a better model membrane system (i.e., unilamellar vesicles), thus exploring a more physiologically relevant situation.

Therefore, it can be hypothesized that the αSyn–membrane interaction not only modulates the protein’s conformation and aggregation propensity, but it also provides a system for trapping and silencing the metal-mediated potentially harmful redox reactivity.

## Figures and Tables

**Table 1:** Fitting results of the experimental EXAFS spectra reported in Figure 3.

	Atom Type	N *	Distance (Å)	2σ^2^ (Å^2^)	R-Factor	χ_ν_^2^	ΔE_0_ (eV)
**αSyn_1–15_ SDS-Cu^+^**	S	2	2.16 (1)	0.003 (1)	0.011	6	−4 (2)
	S	2	2.68 (1)	0.005 (1)			
	C1	4	2.35 (1)	0.013 (1)			
	C2	4	2.44 (1)	0.013 (1)			
**αSyn SDS-Cu^+^**	S	2	2.12 (1)	0.007 (1)	0.042	11	−2 (2)
	S	2	3.00 (1)	0.015 (1)			
**αSyn UVs-Cu^+^**	S	4	2.28 (1)	0.011 (1)	0.022	29	6 (2)

N *—coordination number.

**Table 2:** Relative modification percentage of αSyn fragments in the presence of (a) 4MC and copper(II), (b) 4MC and copper(I) in micellar SDS, and (c) 4MC and copper(II) in SDS micelles. All modifications correspond to the *O*-insertion on the residues listed below, except for the star-marked (*) modification of His50 which represents an increment mass of +120/122 amu.

Time/min	Modified Residues (%)						
	Met1	Met5	His50	Met116	Tyr125	Met127	Tyr133
**(a)**							
**30**	38	85	1	23	19	-	-
**240**	53	72	21 *	72	27	22	29
**(b)**							
**30**	-	-	-	<1	-	-	-
**(c)**							
**30**	-	9	8	<1	-	<1	-

## Data Availability

The data presented in this study are available in insert article or Supplementary Material here.

## Supplementary Materials

The following supporting information can be downloaded at: https://www.mdpi.com/article/10.3390/biom13020287/s1, Figure S1: OverlaidC HSQC spectra of micelles-bound αSyn 240 μM (left panel) and αSyn 450 μM (right panel) in absence and in presence of Ag: 0 eq (black), 0.8 eq (light blue). SDS-d 50mM, phosphate buffer 20 mM pH = 7.4, T = 298 K; Figure S2: Overlaid H 1D spectra of micelles-bound αSyn (lower trace) and acetylated αSyn in absence (black) and in presence of 0.8 Ag eq (magenta). Protein concentrations 240 μM, SDS-d 50mM, phosphate buffer 20 mM pH = 7.4, T = 298 K; Table S1: Fitting results of the EXAFS spectra including different models; Figure S3: Far UV CD spectra at 298K of 10 μM full length αSyn at pH 7.4, in the presence of 50 mM SDS. Spectra were recorded at different Cu concentration: 0 eq (black), 1 eq (light gray), 2 eq (dark grey); Table S2: Anisotropic magnetic parameters of micelle bound αSyn, αSyn and αSyn copper(II) complexes; Figure S4: Comparison of EPR spectra of micelle bound αSyn Cu complex (red line) and hexaaquacopper(II) ion (black line). αSyn 500 μM, Cu μM, SDS 50mM, phosphate buffer 20 mM pH = 7.4; Figure S5: Overlaid H-H TOCSY (A) and H-C HSQC (B) spectra of αSyn before (sea-green) and after the addition of increasing Cu equivalent: 0.2 Cu equiv. (magenta), 0.4 Cu equiv. (green), 0.6 Cu equiv. (pink). αSyn 450 μM, SDS-d 50mM, phosphate buffer 20 mM pH = 7.4, T = 298 K; Figure S6: CD spectra of Cu/Ag αSyn micelle bound system. αSyn 450 μM, SDS 50mM, phosphate buffer 20 mM pH = 7.4, T = 298 K; Figure S7: Kinetic profiles of 4-MC (3 mM) oxidation with time in 50 mM HEPES buffer at pH 7.4 and 20 °C in the presence of Cu (25 μM) without SDS (black trace) and with SDS (20 mM) (orange), and with the addition of αSyn (50 μM, left panel and 25 μM, right panel) without SDS (brown) and with SDS (20 mM) (grey); Figure S8: Kinetic profiles of dopamine (3 mM) oxidation with time in 50 mM HEPES buffer at pH 7.4 and 20 °C in the presence of Cu (25 μM) (black trace) and upon the addition of 25 (orange) and 50 μM (grey) αSyn in buffered solution alone (solid traces) or in SDS micelles (dotted traces); Figure S9: MS/MS spectra of more relevant fragments containing an insertion of one O-atom or of a catechol molecule obtained from HPLC-MS analysis of pepsin-digested αSyn in the reaction mixture. The assignments of the y and b ion series is shown. At the top of each spectrum, the residues which have undergone the modification are shown as star-marked and the summary of the y and b ions found in the spectrum for each fragment is also presented.
