# Supplementary material for: Copper Binding and Redox Activity of α-Synuclein in Membrane-Like Environment
Source: Biomolecules. 2023 Feb 3;13(2):287. doi: 10.3390/biom13020287 (PMC9953312; doi:10.3390/biom13020287)
Supplement: Supplementary file 1 [file biomolecules-13-00287-s001.zip › biomolecules-2166651-supplementary.pdf]

Supporting Information

Copper binding and redox activity of α-Synuclein in membrane like environment

Chiara Bacchella[1], Francesca Camponeschi[2], Paulina Kolkowska[2], Arian Kola[2], Isabella Tessari[3], Maria Camilla Baratto[2], Marco Bisaglia[3,4], Enrico Monzani[1], Luigi Bubacco[3,4], Stefano Mangani[2] , Luigi Casella[1], Simone Dell'Acqua[1,*] and Daniela Valensin[2,5,*]

[1]Department of Chemistry, University of Pavia, Via Taramelli 12, 27100 Pavia, Italy

[2]Department of Biotechnology. Chemistry and Pharmacy, University of Siena, Via Aldo Moro 2, 53100 Siena, Italy

[3] Department of Biology, University of Padova, Padua 35121, Italy

[4]Study Center for Neurodegeneration (CESNE), Padua 35121, Italy.

[5]CIRMMP, Via Luigi Sacconi 6, 50019 Sesto Fiorentino (FI), Italy.

*Correspondence: D.V. daniela.valensin@unisi.it; Tel.+390577232428. S.D. simone.dellacqua@unipv.it; Tel.+390382987354

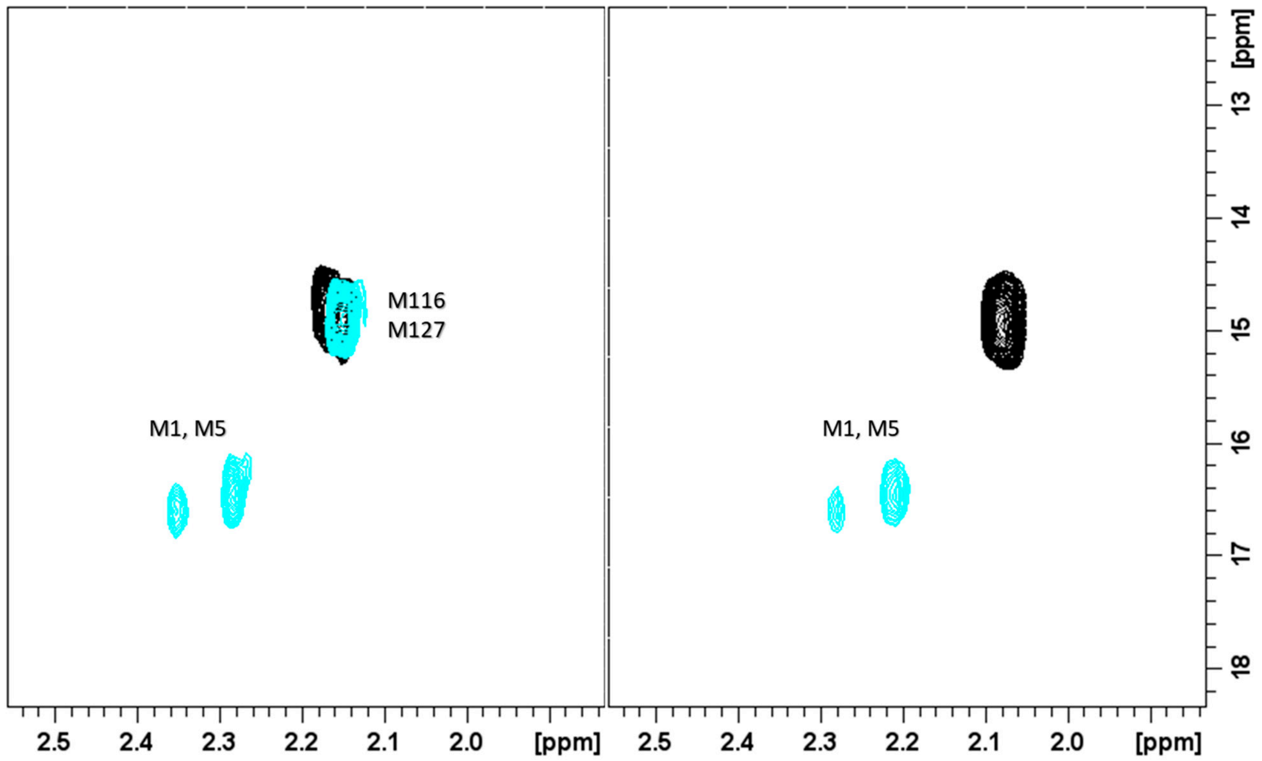

Figure S1. Overlaid ^{1}H-^{13}C HSQC spectra of micelles-bound αSyn$_{1-140}$ 240 µM (left panel) and αSyn$_{1-15}$ 450 µM (right panel) in absence and in presence of Ag^{+}: 0 eq (black), 0.8 eq (light blue). SDS-d$_{25}$ 50mM, phosphate buffer 20 mM pH= 7.4, T =298 K.

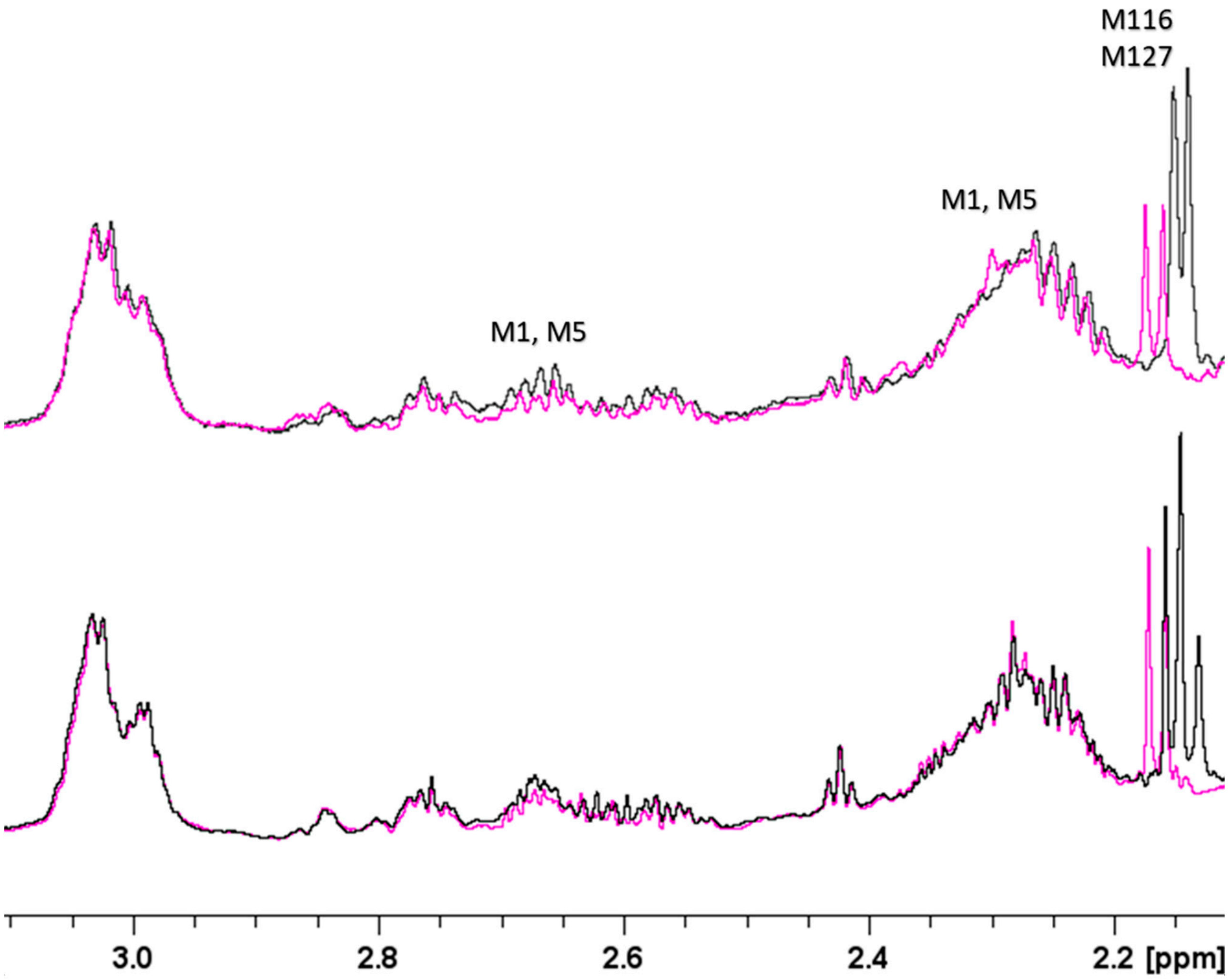

Figure S2. Overlaid ^{1}H 1D spectra of micelles-bound αSyn$_{1\text{-}140}$ (lower trace) and acetylated αSyn$_{1\text{-}140}$ in absence (black) and in presence of 0.8 Ag^{+} eq (magenta). Protein concentrations 240 µM, SDS-d_{25} 50mM, phosphate buffer 20 mM pH= 7.4, T =298 K.

Table S1. Fitting results of the EXAFS spectra including different models.

	Nº Fit	Atom type	N*	Distance (Å)	$2\sigma^2$ (Å^2)	R - factor	χ_v^2	ΔE_0 (eV)
**αS$_{1-15}$ SDS-Cu$^+$**	1	S	2	2.16 (1)	0.002 (1)	0.023	12	-5 (3)
		S	2	2.68 (1)	0.005 (1)			
	2	S	4	2.22 (1)	0.008 (1)	0.066	54	3 (4)
	3	N/O	1	2.05 (1)	0.003 (1)	0.039	32	8 (4)
		S	3	2.24 (1)	0.006 (1)			
	4	S	3	2.23 (1)	0.004 (1)	0.033	27	5 (3)
	5	N/O	1	2.05 (1)	0.001 (1)	0.034	28	10 (4)
		S	2	2.25 (1)	0.002 (1)			
**αSyn SDS-Cu$^+$**	1	S	2	2.11 (1)	0.007 (1)	0.041	11	-3(3)
		S	1	2.99 (1)	0.013 (1)			
	2	N/O	1	1.94 (1)	0.002 (1)	0.045	12	5 (3)
		S	1	2.15 (1)	0.005 (1)			
		S	1	3.04 (1)	0.007 (1)			
	3	S	3	2.10 (1)	0.012 (2)	0.078	20	-6 (4)
	4	S	1	2.12 (2)	0.001 (2)	0.107	27.5	0 (5)
		S	1	2.99 (2)	0.002 (2)			
	5	S	2	2.11 (1)	0.007 (1)	0.063	16	-5 (4)
	6	S	4	2.09 (2)	0.017 (2)	0.125	32	-9 (5)
**αSyn UV-Cu$^+$**	1	S	3	2.27 (1)	0.008 (1)	0.026	36	6 (3)
	2	N/O	1	2.06 (1)	0.005 (1)	0.060	62	5
		S	3	2.25 (1)	0.009 (1)			
	3	N/O	1	2.06 (1)	0.003 (1)	0.097	100	5
		S	2	2.25 (1)	0.005 (1)			
	4	S	2	2.21 (2)	0.005 (2)	0.066	89	-2 (6)
		S	2	2.74 (2)	0.010 (2)			

N – coordination number.*

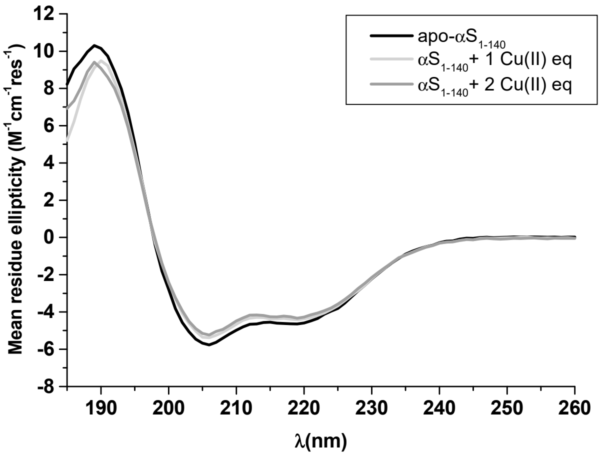

Figure S3. Far UV CD spectra at 298K of 10 μM full length αSyn at pH 7.4, in the presence of 50 mM SDS. Spectra were recorded at different Cu^{2+} concentration: 0 eq (black), 1 eq (light gray), 2 eq (dark grey).

Table S2. Anisotropic magnetic parameters of micelle bound αSyn_{1-140}, αSyn_{1-15} and αSyn_{45-15} copper(II) complexes.

	$g_{\|\|}$[a]	$g_{\perp}$[a]	$A_{\|\|}$(mT)[b]	$A_{\perp}$(mT)[b]	n°N[c]	A_N(mT)[b]
**αSyn_{1-140}**	2.236	2.056	17.8	0.5	2	1.4
**αSyn_{1-15}**	2.247	2.058	18.0	0.65	2	8
**αSyn_{45-55}**	2.2	2.076	122	7.5	-	-

[a] *Estimated error ±0.001.* [b] *Coupling constant are given in mT. Estimated error for* A_{Cu} = *±0.1mT and for* A_N = *±0.05mT.* [c] *Number of equatorial nitrogen donor atoms.*

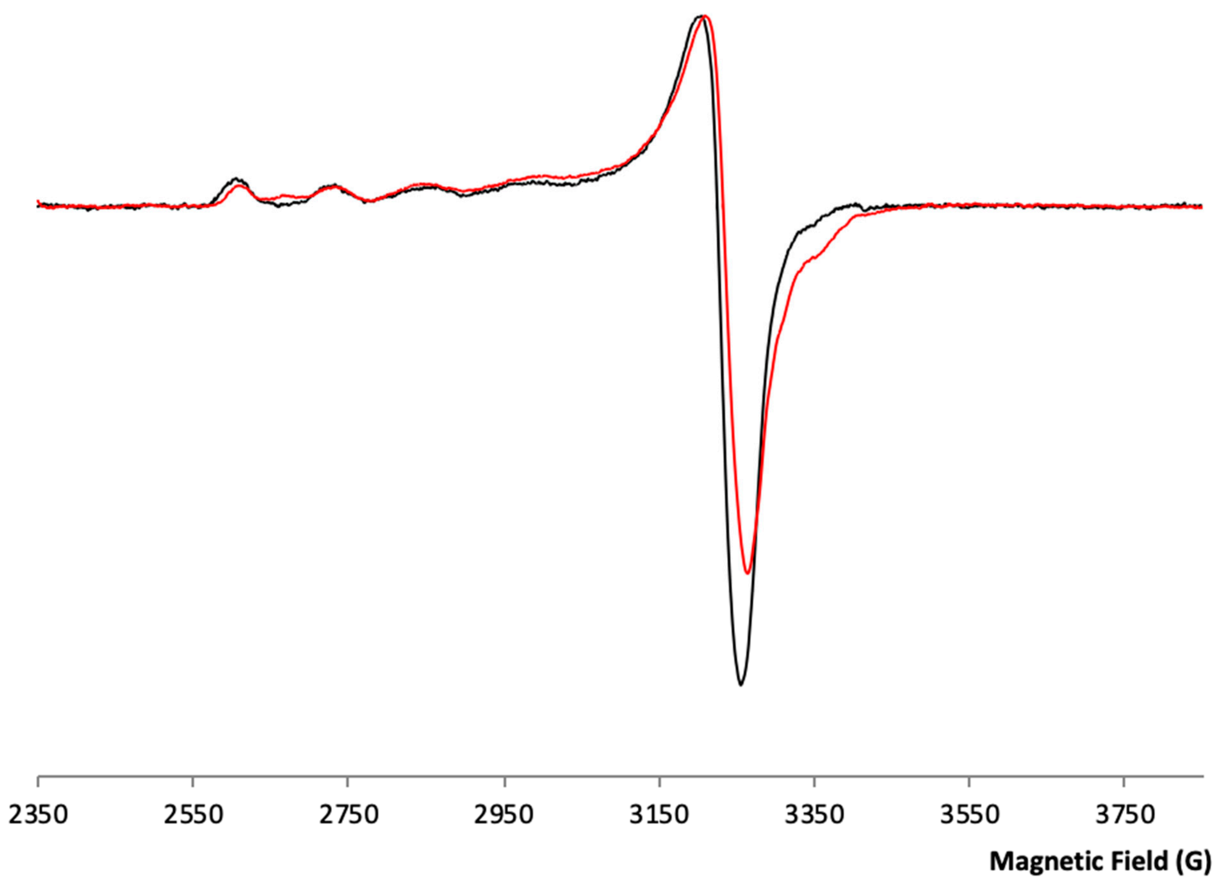

Figure S4. Comparison of EPR spectra of micelle bound αSyn$_{45\text{-}55}$ Cu^{2+} complex (red line) and hexaaquacopper(II) ion (black line). αSyn$_{45\text{-}55}$ 500 µM, Cu^{2+} µM, SDS 50mM, phosphate buffer 20 mM pH= 7.4.

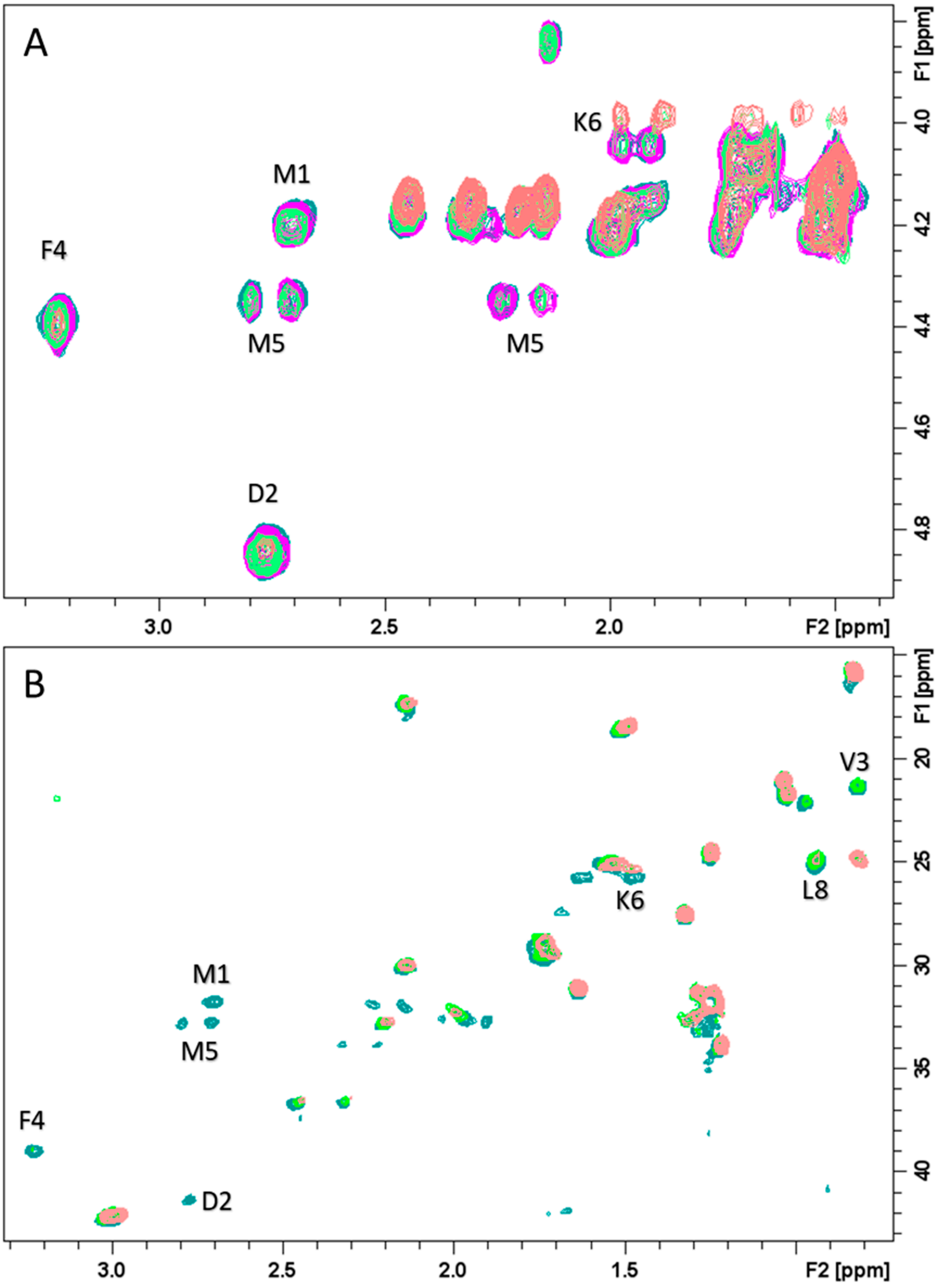

Figure S5. Overlaid ^{1}H-^{1}H TOCSY (A) and ^{1}H-^{13}C HSQC (B) spectra of αSyn_{1-15} before (sea-green) and after the addition of increasing Cu^{2+} equivalent: 0.2 Cu^{2+} equiv. (magenta), 0.4 Cu^{2+} equiv. (green), 0.6 Cu^{2+} equiv. (pink). αSyn_{1-15} 450 µM, SDS-d_{25} 50mM, phosphate buffer 20 mM pH= 7.4, T =298 K.

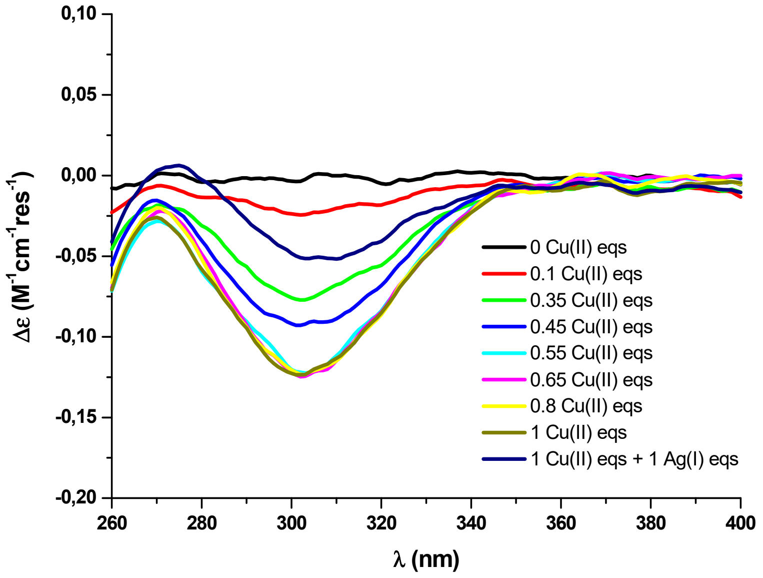

Figure S6. CD spectra of Cu^{2+}/Ag^{+} αSyn_{1-15} micelle bound system. αSyn_{1-15} 450 μM, SDS 50mM, phosphate buffer 20 mM pH= 7.4, T =298 K.

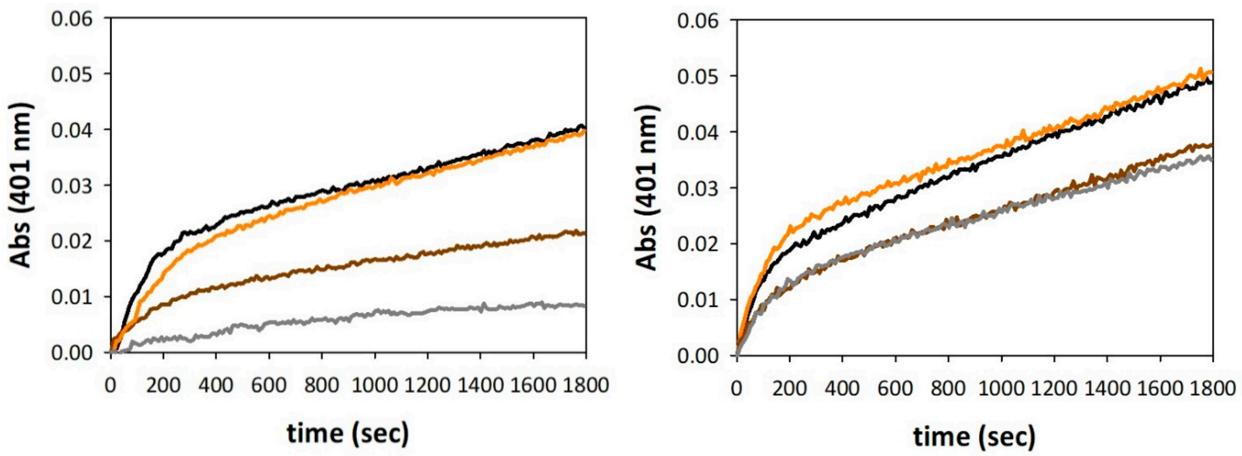

Figure S7. Kinetic profiles of 4-MC (3 mM) oxidation with time in 50 mM HEPES buffer at pH 7.4 and 20 °C in the presence of Cu^{2+} (25 µM) without SDS (black trace) and with SDS (20 mM) (orange), and with the addition of αSyn (50 µM, left panel and 25 µM, right panel) without SDS (brown) and with SDS (20 mM) (grey).

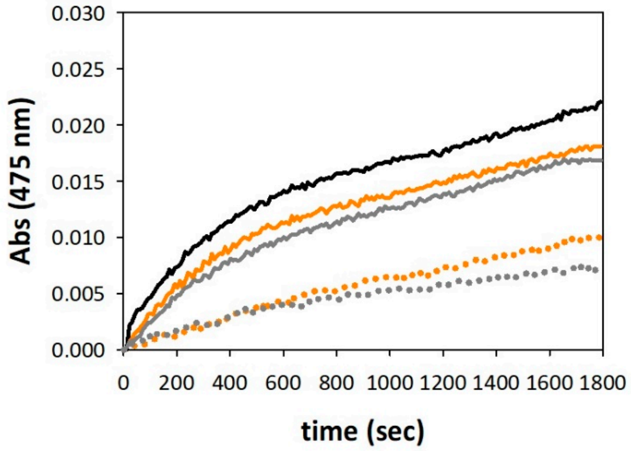

Figure S8. Kinetic profiles of dopamine (3 mM) oxidation with time in 50 mM HEPES buffer at pH 7.4 and 20 °C in the presence of Cu^{2+} (25 µM) (black trace) and upon the addition of 25 (orange) and 50 µM (grey) αSyn in buffered solution alone (solid traces) or in SDS micelles (dotted traces).

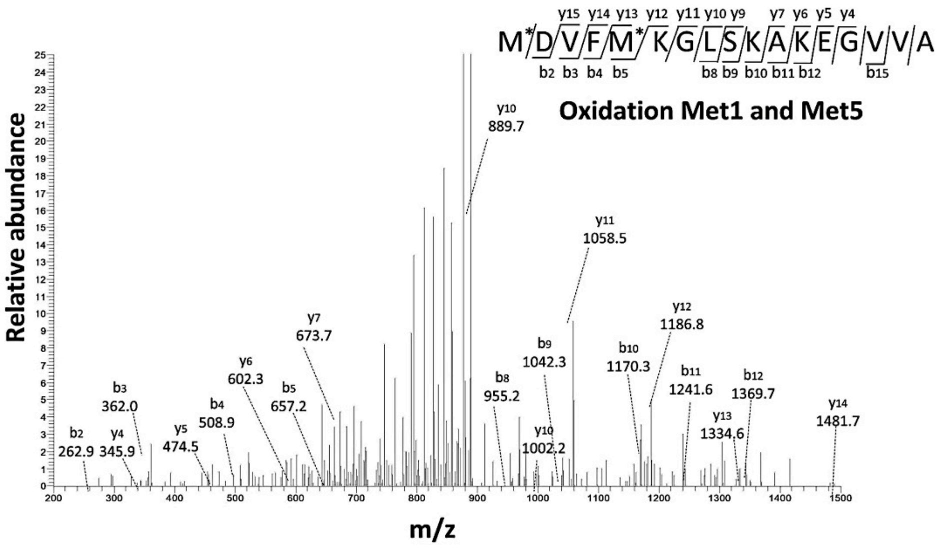
M*DVFM*KGLSKAKEGVVA
Oxidation Met1 and Met5
y15 y14 y13 y12 y11 y10 y9 y7 y6 y5 y4
b2 b3 b4 b5 b8 b9 b10 b11 b12 b15
y10 889.7
y11 1058.5
y12 1186.8
y7 673.7
b9 1042.3
b10 1170.3
b3 362.0
y6 602.3
b5 657.2
b8 955.2
b11 1241.6
b12 1369.7
b4 508.9
y10 1002.2
y13 1334.6
y14 1481.7
b2 262.9
y4 345.9
y5 474.5
Relative abundance
m/z

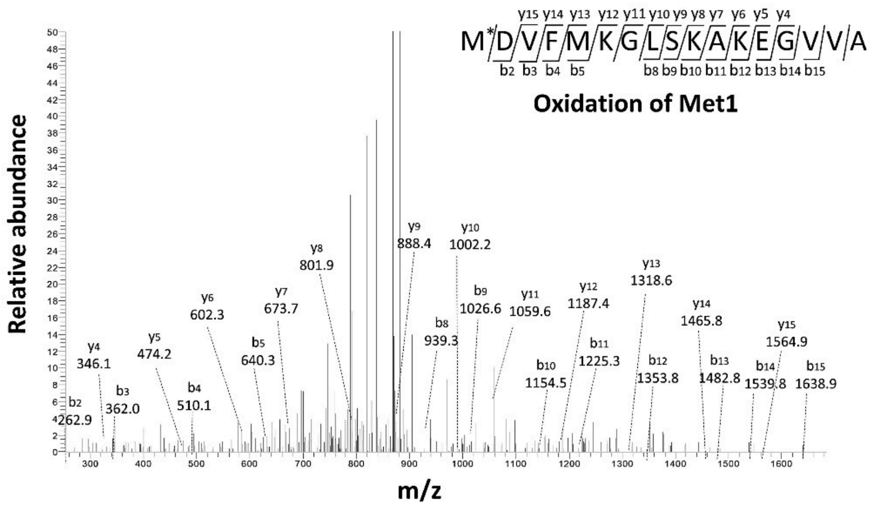
M*DVFMKGLSKAKEGVVA
Oxidation of Met1
y15 y14 y13 y12 y11 y10 y9 y8 y7 y6 y5 y4
b2 b3 b4 b5 b8 b9 b10 b11 b12 b13 b14 b15
y9 888.4
y10 1002.2
y8 801.9
y13 1318.6
y6 602.3
y7 673.7
b9 1026.6
y11 1059.6
y12 1187.4
y14 1465.8
y15 1564.9
b8 939.3
y4 346.1
y5 474.2
b5 640.3
b11 1225.3
b10 1154.5
b12 1353.8
b13 1482.8
b14 1539.8
b15 1638.9
b2 262.9
b3 362.0
b4 510.1
Relative abundance
m/z

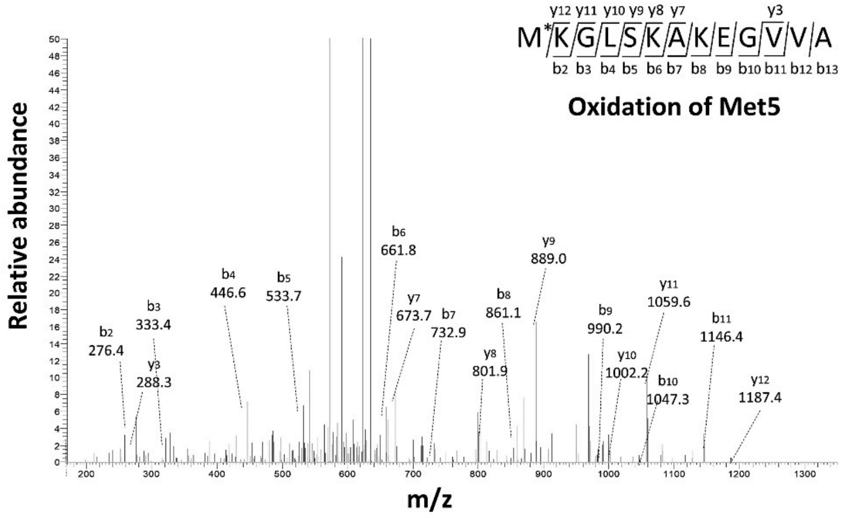
M*KGLSKAKEGVVA
Oxidation of Met5
y12 y11 y10 y9 y8 y7 y3
b2 b3 b4 b5 b6 b7 b8 b9 b10 b11 b12 b13
b6 661.8
y9 889.0
b4 446.6
b5 533.7
y7 673.7
b7 732.9
b8 861.1
y11 1059.6
b3 333.4
b2 276.4
y3 288.3
b9 990.2
b11 1146.4
y10 1002.2
y8 801.9
b10 1047.3
y12 1187.4
Relative abundance
m/z

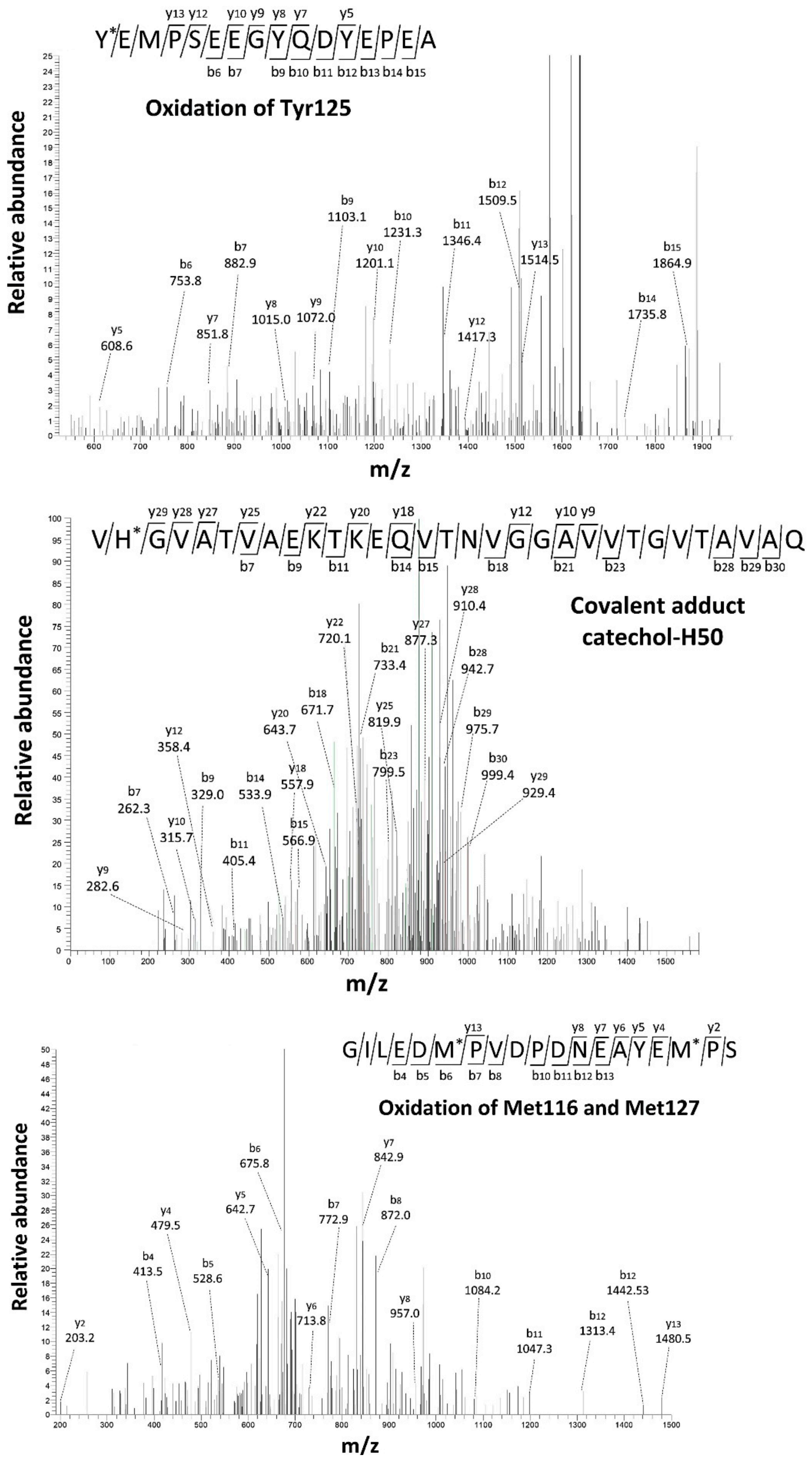

Figure S9. MS/MS spectra of more relevant fragments containing an insertion of one O-atom or of a catechol molecule obtained from HPLC-MS analysis of pepsin-digested αSyn in the reaction mixture. The assignments of the y and b ion series is shown. At the top of each spectrum, the residues which have undergone the modification are shown as star-marked and the summary of the y and b ions found in the spectrum for each fragment is also presented.
